# Successful Treatment of Posttransplant EBV-Associated Lymphoma and Plasmacytoma Solely Localized to the CNS

**DOI:** 10.1155/2012/497614

**Published:** 2012-02-28

**Authors:** Per Boye Hansen, Signe Ledou Nielsen

**Affiliations:** ^1^Department of Hematology, Herlev Hospital, University of Copenhagen, Copenhagen, 2730 Herlev, Denmark; ^2^Department of Pathology, Herlev Hospital, University of Copenhagen, Copenhagen, 2730 Herlev, Denmark

## Abstract

Two patients with diabetic nephropathy were diagnosed with primary central nervous system posttransplant *Epstein-Barr-virus*-associated lymphoproliferative disorder (PTLD) 3 years after renal transplantation. The histological diagnoses of the isolated brain tumors were diffuse large B-cell lymphoma and plasmacytoma. Considerable co-morbidity precluded intensive chemotherapy. The first patient with lymphoid CD20+ PTLD had a partial resection of her tumor performed. She was treated with 4 weekly doses of rituximab, ganciclovir and prednisolone; the posttransplant immune suppression (tacrolimus) was reduced. After 4 weeks of treatment a magnetic resonance imaging (MRI) demonstrated complete regression of the CNS lesion. The patient continues to receive rituximab (every second month), valgangciclovir and low-dose prednisolone. Twenty-two months after initiation of therapy, she is still in complete remission. The second patient was only treated with craniospinal irradiation involving the medulla to the second cervical vertebra and valgangciclovir. Moreover, the posttransplant immune suppression was reduced. A new MRI two months after initiation of therapy showed a complete regression of the lesions in the CNS; this was again demonstrated by a MRI after 19 months. These 2 cases illustrate interesting alternative treatments of PTLD. To our knowledge, an EBV-associated PTLD of plasmacytic origin isolated to the CNS has never been described before.

## 1. Introduction

Posttransplant lymphoproliferative disorders (PTLDs) are rare lymphoid or plasmacytic proliferations, which occur following allogeneic hematopoietic stem cell transplantation or solid organ transplantation. PTLDs are characteristically aggressive with a rapid onset and are often localized in extranodal sites, such as the gastrointestinal tract, lungs, liver, and kidney, whereas central nervous system (CNS) involvement is rare. They are mostly of B-cell origin, and about 90% are associated with *Epstein-Barr virus* (EBV) infection, with EBV genome detected in the malignant cells [1]. The proposed pathogenesis includes EBV reactivation in B-lymphocytes of immunosuppressed patients, leading to uncontrolled monoclonal B-lymphocyte proliferation and transformation to malignant cells.

Because of its rarity, there is no consensus on the optimal treatment for PTLD, and no large prospective phase III trials have been published. However, treatment strategies in adult solid organ transplant recipients include a reduction or withdrawal of immunosuppressive therapy—which alone may lead to partial or complete regression in a proportion of cases—surgery, radiotherapy, antiviral drugs, combination chemotherapy, and monoclonal antibodies directed against B-cell CD20 (rituximab) [2–4]. In cases of PTLD following hematopoietic stem cell transplantation, infusion of donor-derived EBV-specific cytotoxic T cells has shown some efficacy [5]. In the following, we describe two patients with EBV PTLD following solid organ transplantation with isolated CNS involvement. One patient with lymphoma was successfully treated with rituximab, antiviral treatment, and prednisolone (first case); the other patient with plasmacytoma received CNS radiation and antiviral treatment (second case).

## 2. Case Presentations

### 2.1. Case

**Figure 1:**
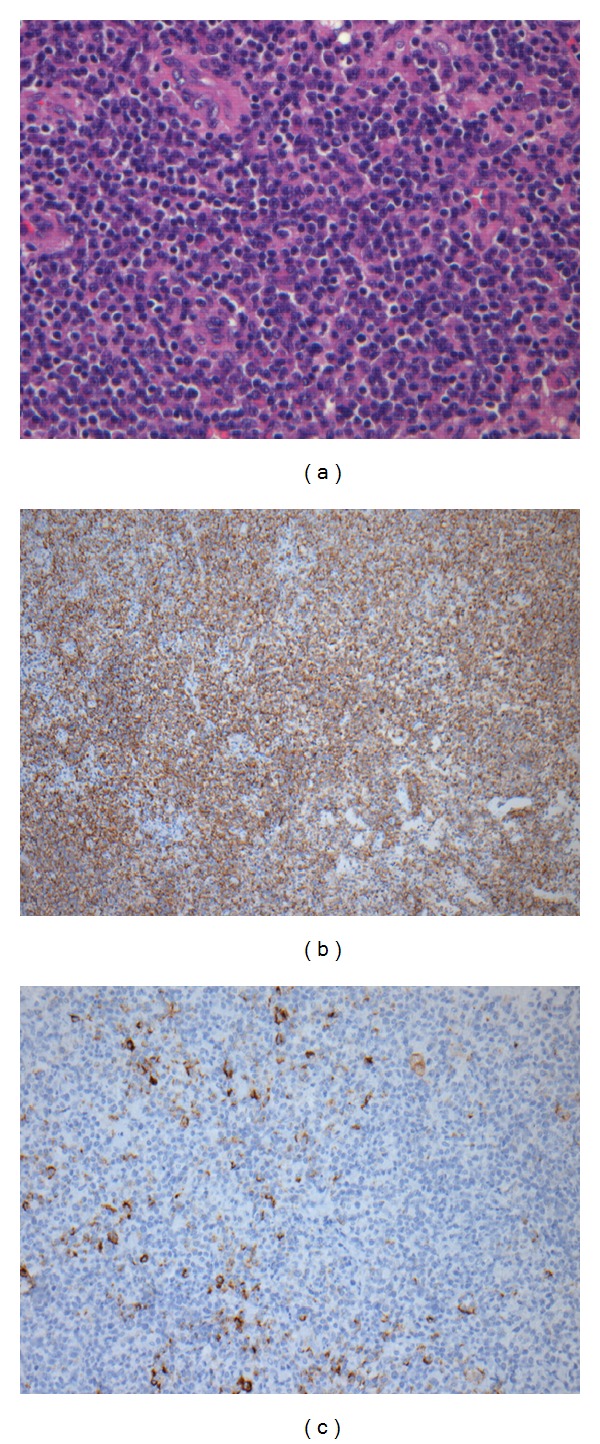
Histological and immunohistochemical analysis of posttransplant EBV-associated diffuse large B-cell lymphoma in the CNS. (a) H&E section showing diffuse infiltration by large neoplastic lymphocytes (×20). (b) Immunohistochemistry showing neoplastic lymphocytes are strongly positive for CD20 (×20). (c) Immunohistochemistry showing scattered tumor cells positive for EBV (×20).

**Figure 2:**
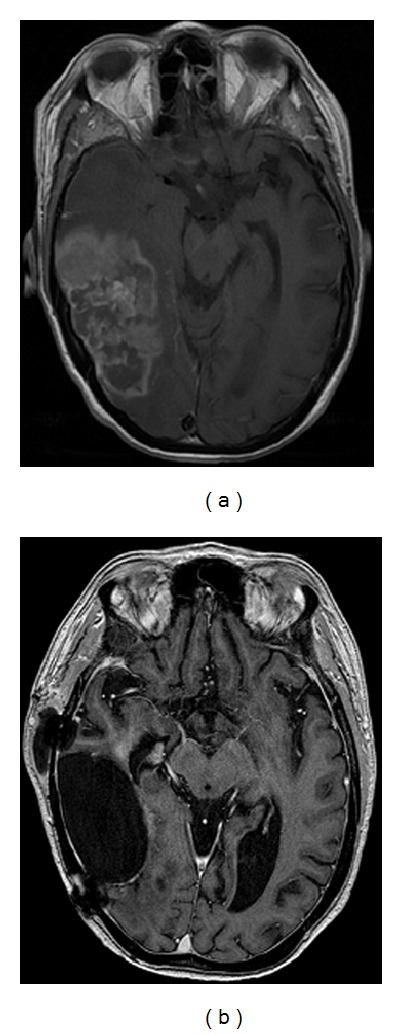
(a) Axial contrast-enhanced MR image of the cerebrum showing a 3.8 × 7.8 cm tumor infiltrating the parietooccipital region of the right hemisphere. The lesion is with surrounding edema and compressing the right lateral ventricle and the right side of the mesencephalon. (b) Four weeks after start of treatment, a resection cavity with a modest enhancement against the calvaria is seen. The centre line is in normal position, and the content of the cavity is isodense with the cerebrospinal fluid.

A 35-year-old female was transferred to the department of hematology with a primary central nervous system posttransplant EBV*-*associated lymphoproliferative disorder. Biopsy from the tumor showed a highly necrotic polymorphous infiltrate of variably sized lymphocytes. The large cells displayed pleomorphic nuclei, with prominent nucleoli, some of which had immunoblastic appearance (Figure 1(a)). Immunohistochemical studies showed the large atypical cells to be positive for CD20 (Figure 1(b)). The histological diagnosis of the tumor was diffuse large B-cell lymphoma, and EBV was detected in the tumor cells by immunohistochemistry for latent membrane protein-1 (LMP-1) (Figure 1(c)). The patient had been diagnosed with diabetes mellitus type 1 at age 7; a renal transplantation was performed 3 years earlier because of diabetic nephropathy. After the transplantation, the patient was initially treated with cyclosporine, and, after 6 months, the immunosuppressive therapy was changed to tacrolimus (4 mg twice a day). Prior to the admission, the patient had suffered from headaches, nausea, and dizziness for several weeks. A magnetic resonance imaging (MRI) of the CNS showed a 3.8 × 7.8 cm tumor infiltrating the right parietooccipital region; there was surrounding edema and compression of the right lateral ventricle and right side of the mesencephalon (Figure 2(a)). A complete resection was not possible, and treatment with prednisolone, 100 mg per day, was started. A bone marrow examination and computer tomography (CT) of the neck, chest, and abdomen did not show lymphoma outside the CNS. HIV testing was negative. An MRI 3 days after the operation showed a 3 × 6 cm resection cavity with contrast in the remaining tumor and nonresolved compression of the ventricle system. Obviously, intrathecal therapy was not possible, and, due to considerable comorbidity, intensive chemotherapy and/or radiation therapy was not feasible. Instead, the patient was treated with the monoclonal antibody rituximab, ganciclovir, and continuous high-dose prednisolone; the immune suppression was reduced in close cooperation with the department of nephrology (tacrolimus 1 mg twice a day). Rituximab was given in 4 weekly doses of 500 mg/m^2^, the antiviral drug ganciclovir (5 mg/kg i.v. twice a day for 14 days) followed by valgangciclovir (450 mg by mouth every second day). After 4 weeks of treatment, an MRI demonstrated a complete regression of the CNS lesion and resolution of the displacement of the ventricular system (Figure 2(b)). The patient was discharged in good condition, without neurological defects, and is still in complete remission twenty-two months later. In the outpatient clinic, the patient is receiving rituximab (500 mg/m^2^) every second month and is on continuous treatment with valgangciclovir (450 mg by mouth every second day).

### 2.2. Case

**Figure 3:**
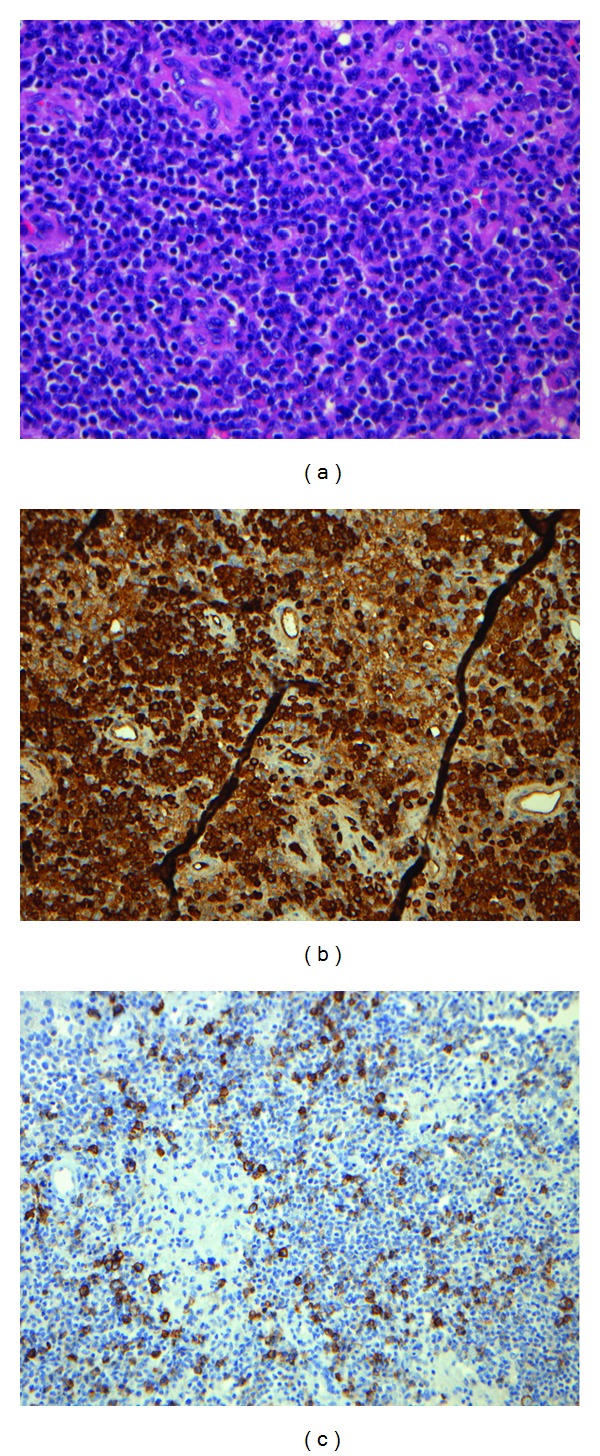
Histological and immunohistochemical analysis of posttransplant EBV-associated plasmacytoma in the CNS. (a) H&E section of a tumor showing a solid infiltrate of atypical malignant plasma cells (×40). (b) The plasma cells are positive for lambda light chains by immunophenotyping (×20). (c) Immunohistochemistry showing scattered tumor cells positive for EBV (×20).

A 53-year-old male was admitted with a history of mental confusion, headaches, dysarthria, and ataxia of a few weeks duration. The patient had been diagnosed with diabetes mellitus type 1 at age 17; a renal transplantation was performed in 2006 due to diabetic nephropathy. The posttransplant treatment consisted of immunosuppression with tacrolimus and mycophenolate mofetil. An MRI of the brain showed 4 solid lesions in the cerebral hemispheres with surrounding edema. A stereotactic brain biopsy was performed; this revealed atypical, malignant plasma cells (Figure 3(a)). The plasma cells were monoclonal for lambda light chains by immunophenotyping (Figure 3(b)), and EBV genome was detected in the tumor cells by immunohistochemistry for LMP-1 (Figure 3(c)). A lumbar puncture did not reveal malignant clonal cells by cytological examination of the cerebrospinal fluid (CSF), and flow cytometry was normal. A PET/CT and bone marrow examination eliminated multiple myeloma, and the diagnosis PTLD with an isolated cerebral plasmacytoma was made. Due to his poor condition, the patient was only treated with high-dose prednisolone (150 mg/day), reduction of the immunosuppressive treatment, craniospinal irradiation to the second cervical vertebra (2  gray × 20), and oral antiviral treatment with valgangciclovir (900 mg by mouth twice a day for 21 days, followed by 450 mg every second day because of the renal function). An MRI two and 19 months later showed a complete regression of the CNS lesions. Unfortunately, the patient died three weeks after the last MRI due to diabetic complications. Autopsy was not performed.

## 3. Discussion

PTLD is a special subtype of lymphoproliferative disorders and a rare complication of immunosuppression following solid organ, bone marrow, or stem cell allogenic transplantation. However, the incidence is increasing with a frequency varying with the intensity of the immunosuppressive regimen. The lowest incidence is after renal transplantation (<1%) and the highest after heart/lung and intestinal transplantation (≥5%) [6, 7]. There is no gold standard for the treatment of PTLD, but as the association with EBV is well described, and the virus is actively responsible for the B-cell proliferation at the time of diagnosis, reduction of immunosuppression is a logical treatment approach. The localization of PTLD in the CNS represents a therapeutic problem as only a few cytostatic drugs are able to cross the blood-brain barrier effectively. Moreover, the efficacy of systemic treatment with rituximab in CNS diseases is debatable, since rituximab has a high molecular weight and crosses the intact barrier very poorly. In pharmacokinetic studies, the concentration in the cerebrospinal fluid was approximately 0.1% compared to the value in the serum [8]. In a prospective multicenter phase 2 study, monotherapy with rituximab was given to patients with PTLD outside the CNS, who were not responding to tapering of immunosuppression [3]. The treatment consisted of rituximab given in 4 weekly doses of 375 mg/m^2^. The response rate was 44.2%, and 67% of the patients were alive after 1 year. Due to these results and the results of a retrospective study [9], preemptive treatment with rituximab is recommended in high-risk patients with an increasing EBV load in the peripheral blood.

PTLD in the CNS is very rare and is only described as case reports in the literature [10, 11]. In two cases, the EBV PTLD was only localized to the CNS and was successfully treated with rituximab [10]. In another case, with EBV PTLD in peripheral regions and CNS, a complete remission was obtained during treatment with rituximab and the antiviral drug cidofovir [11]. These cases, combined with our results, may suggest that the blood-brain barrier is deficient in PTLD in the CNS, perhaps due to abnormal tumor vessels and the edema that often surrounds the lymphoma. Rituximab combined with antiviral therapy, and if possible reduction of immunosuppression, is a nontoxic alternative to systemic and intrathecal chemotherapy in this group of patients. Cerebral involvement in multiple myeloma is very uncommon with a poor prognosis. In two studies with review of the literature, the median survival from the time of diagnosis to death is 1.5 and 2.0 months, respectively [12, 13]. In a case report, an intracerebral plasmacytoma has been diagnosed as the initial presentation of multiple myeloma [14], but, to our knowledge, an EBV PTLD of plasmacytic origin with exclusive CNS involvement has not been described previously. Moreover, our patient had a much more indolent clinical course than patients with multiple myeloma and involvement of the CNS.

90–95% of adults worldwide have been infected with EBV, and PTLD is, with increasing incidence, the most common cause of cancer-related mortality after solid organ transplantation. Due to this, there is a need for randomized phase III trials to define the optimal treatment of this tumor, where the goal is complete remission of the PTLD and preservation of the transplanted organ, while minimizing treatment-related toxicity in this group of patients, who are characterized by considerable serious comorbidity.
